# Anti‐Inflammatory Action and Molecular Mechanism of Fucoidan Against Cystitis Glandularis

**DOI:** 10.1002/fsn3.4560

**Published:** 2024-11-04

**Authors:** Qingting Chen, Jie Mo, Yu Li, Li Gao, Ka Wu

**Affiliations:** ^1^ The Third Affiliated Hospital of Guangxi Medical University, The Second People's Hospital of Nanning City Nanning China; ^2^ Guilin Medical University, Guilin Medical University Guilin China; ^3^ Department of Urology Surgery The Second Affiliated Hospital of Guilin Medical University, Guilin Medical University Guilin China

**Keywords:** computational analysis, cystitis glandularis, fucoidan, pharmacological targets

## Abstract

Cystitis glandularis (CG), known as a pre‐gradual lesion in the bladder, is the pathological changes in the vesical mucosa characterized by inflammatory invasion and chronic obstruction. Clinically, effective treatment against CG is prescribed only when using drug therapy. Fucoidan, the naturally extractive polysaccharide, is well‐reported bioactive compound with anti‐inflammatory and immunoregulatory properties. In this research, an emerging computational approach was applied to explicate anti‐CG actions and pharmacological targets exhibited by fucoidan in detail. Current network pharmacology data showed that 16 intersection genes of fucoidan and CG were identified, whereas all 6 core targets, including interleukin‐6 (IL‐6), tumor necrosis factor (TNF), interleukin‐1B (IL‐1B), matrix metalloproteinase‐9 (MMP‐9), interleukin‐10 (IL‐10), matrix metalloproteinase‐2 (MMP‐2), biological processes, and signaling pathways of fucoidan against CG were characterized, respectively. As revealed in the underlying mechanism, the anti‐CG actions achieved by fucoidan were chiefly implicated in the reduction of inflammatory reactions and enhancement of immunoregulation. Taken together, these network bioinformatics findings may be used to reveal anti‐CG effects and the pharmacological mechanism of fucoidan before further experimental validation. Furthermore, those core genes identified may be therapeutic targets for research and development of fucoidan‐anti‐CG.

## Graphical abstract

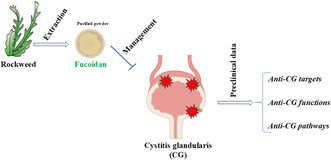

## 1 Introduction

CG, detected with the lesions including cyst formation, epithelial hyperplasia, glandular metaplasia of goblet cells, and urothelium, is a relatively rare non‐neoplastic inflammatory disease (Abdel Magied, Badreldin, and Leslie 2024). Medically, it is speculated controversially that CG may be a possible precursor of cancer development despite clinical evidence being limited (Yi et al. 2014). Nevertheless, CG should be treated by using clinical regimes, as CG may induce sharp pain, odynuria, and vesicovaginal fistulas for severely threatening patients’ health (Ronghua et al. 2024). In medical diagnosis, CG patients are histopathologically identified and accompanied with ultrasound, computed tomography (CT), and conventional magnetic resonance imaging (MRI) tests (Wang et al. 2016). Currently existing treatment, the clinical regimes can have transurethral resection and postoperative intravesical chemotherapy, including cyclooxygenase inhibitors (Bai, Chen, and Zeng 2023). However, clinical medication treating CG is limitedly to be prescribed as it is a refractory disease characterized with poor therapeutic effectiveness and significant recurrence (Takizawa et al. 2016). Thus, specially screening natural ingredients and identifying the pharmacological activities of them may be a promising strategy against CG. Marine algae can be used for extracting anti‐inflammatory compounds, including flavonoids, peptides, and polysaccharides (Ghallab et al. 2024). In preclinical evaluation, marine algae may be used for the cytoprotection of hemorrhagic cystitis, such as spirulina (Sinanoglu et al. 2012). Fucus, a brown algae with antioxidant effects, is found with potential anti‐inflammatory pharmacological properties (Catarino, Silva, and Cardoso 2018). Fucoidan, a bioactive compound rich in Fucus algae, refers to a sulfuric acid polysaccharide complex that exerts antioxidant, anti‐inflammatory, antithrombotic, anticoagulant, antitumor, antiviral actions, and immunoregulation (Zhang et al. 2022). An animal study shows that fucoidan may reduce inflammatory stress in ifosfamide‐induced hemorrhagic cystitis (Dornelas‐Filho et al. 2018). However, the potent pharmacological action and biotarget of fucoidan against CG need to be evaluated and identified. It is increasingly reported that network pharmacology‐based bioinformatics analysis can be used to systematically reveal potential targets and therapeutic mechanisms of bioactive components against clinical disorders (Li et al. 2021; Noor et al. 2022). In addition, our preceding studies highlight that bioinformatics findings by using a network pharmacology approach can be applied for revealing calycosin against osteosarcoma (Pan et al. 2021) and plumbagin against liver cancer (Zhou et al. 2019). In present research, we explained the biological targets and molecular mechanisms of fucoidan action against CG through network pharmacology analysis before future experimental validation, and clinical trials.

## 2 Material and Methods

### 2.1 Acquiring the Fucoidan‐ and CG‐Associated Targets

Methodologically, the bioactive genes of fucoidan were screened and determined in the Comparative Toxicogenomics Database (https://ctdbase.org) after gene symbol annotation by using the Uniprot Knowledgebase (https://www.uniprot.org). Whereafter, GeneCard Suite (www.genecards.org) was employed to screen CG‐associated genes, and the gene set was established by using database retrieval results. All fucoidan‐ and CG‐related gene sets were integrated for identifying intersection targets between fucoidan and CG through Venn diagram analysis (http://bioinformatics.psb.ugent.be/webtools/Venn).

### 2.2 Determining the Protein–Protein Interaction (PPI) Network and Core Targets

All fucoidan‐ and CG‐related gene data sets were employed to construct a PPI network by utilizing the String database (https://string‐db.org/), in which the parameter was set as the minimum required interaction score (0.700). The NetworkAnalyzer analysis from Cytoscape software (https://cytoscape.org/) aimed to check and identify the core targets of fucoidan against CG via the topological arithmetic method, as described elsewhere (Kohl, Wiese, and Warscheid 2011).

### 2.3 Enrichment Analysis for Functional Determination

All target gene sets in core targets were used to produce a compound‐target network by using *R* programming language packages, including “ClusterProfiler,” “org.Hs.eg.Db,” “GOplot.” Enrichment analyses for gene ontology (GO) and Kyoto Encyclopedia of Genes and Genomes (KEGG) were conducted to uncover the pharmacological mechanisms, detailed in biological processes, cellular components, molecular functions, and signaling pathways. The “org.Hs.eg.Db” package was used for gene annotation, and gene enrichment with a *p* value cutoff (0.05) and a *q* value cutoff (0.05) was employed to plot the bubble and circle charts, as described elsewhere (Lu et al. 2023).

### 2.4 Creating the Integrated Bioinformatics Graph

By utilizing Cytoscape software (Version 3.7.1), the drug‐target‐gene‐ontology‐pathway‐disease visualization graph was constructed in detail based on the biological process and pathway enrichment findings of fucoidan against CG.

## 3 Results

### 3.1 Candidate and Mutual Targets of Fucoidan and CG

**FIGURE 1:**
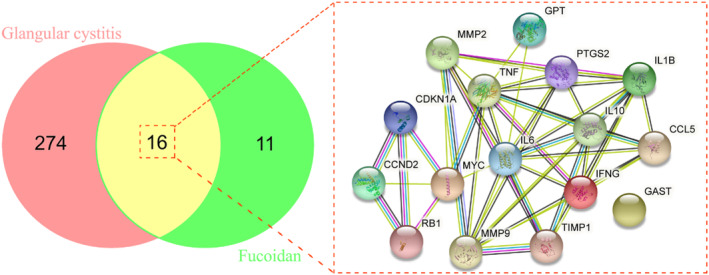
After preliminary network pharmacology analysis, routine and mutual genes of fucoidan and CG were identified, respectively, in a Venn diagram. An association diagram was created for all 16 mutual genes.

Being excluded from the duplication data, a gene set of 27 fucoidan‐related targets was gained, and a total of 290 target genes of CG were obtained accordingly. Moreover, we summarized 16 intersection gene sets between fucoidan and CG that were harvested finally, in which a fucoidan‐CG network diagram interconnecting these intersection genes is visualized in Figure 1.

### 3.2 Core Targets of Fucoidan Against CG

**FIGURE 2:**
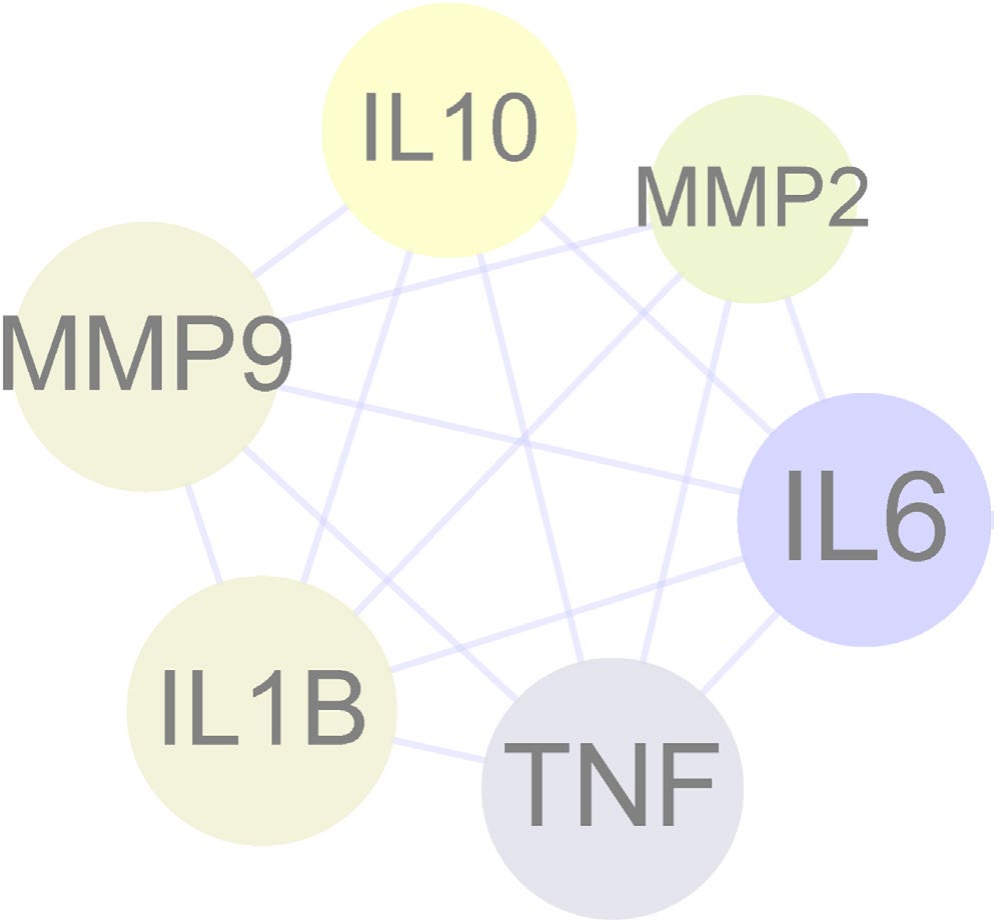
All six core targets of fucoidan against CG, including IL‐6, TNF, IL‐1B, MMP‐9, IL‐10, and MMP‐2, were determined.

Following the topological parameter algorithm, the median of degrees of freedom was set to 6.627, and the maximum degree of freedom was set to 11. Then, the screening criterion range for core targets was set to from 7 to 11. As a result, all core targets of fucoidan against CG were determined, including IL‐6, TNF, IL‐1B, MMP‐9, IL‐10, and MMP‐2 (Figure 2). Other detailed information for core targets is presented in Table S1.

### 3.3 GO‐Based Enrichment Analysis Findings

**FIGURE 3:**
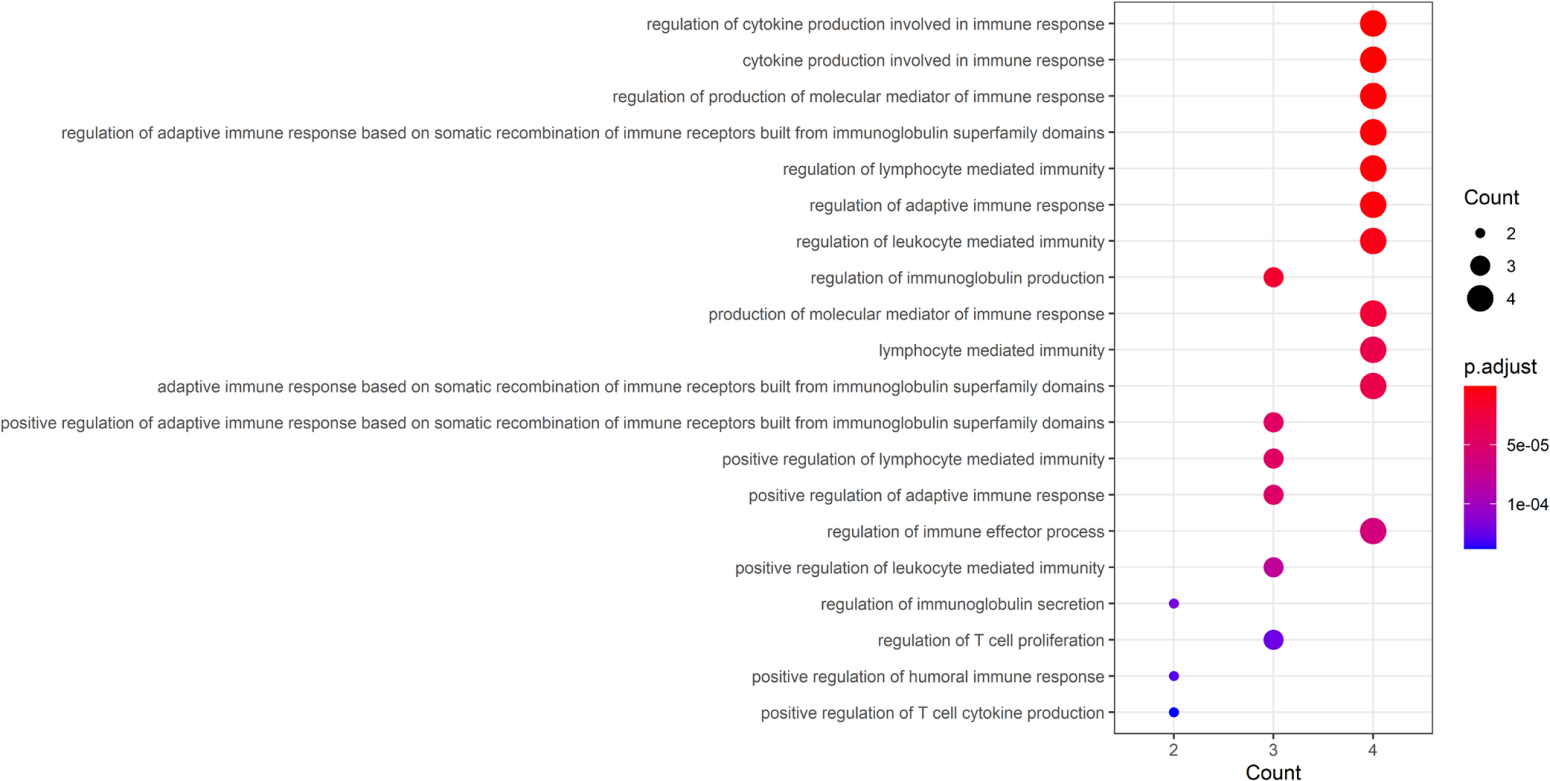
Current GO‐based enrichment analysis revealed these top immunologic function‐related biological processes of fucoidan against CG.

**FIGURE 4:**
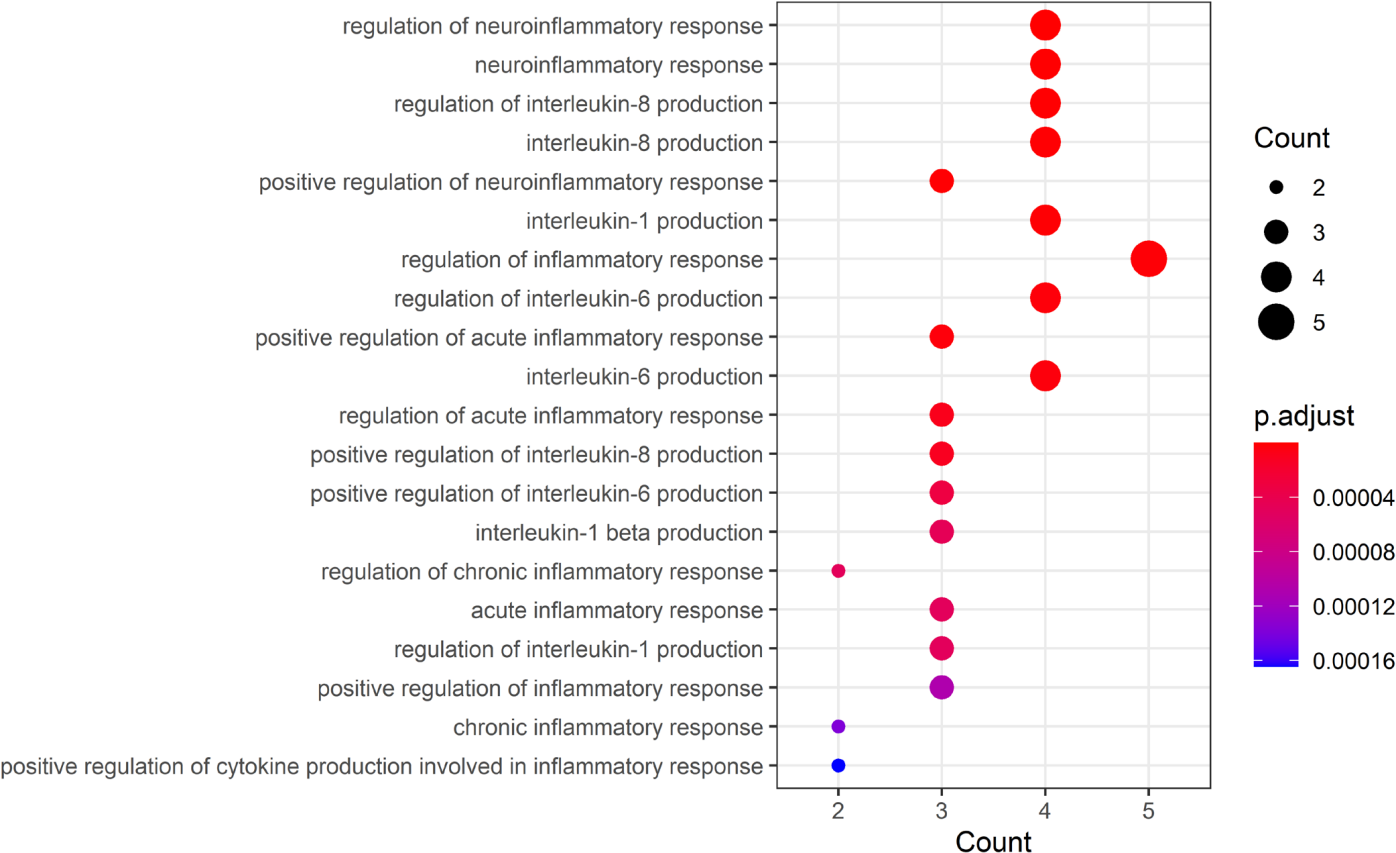
In addition, these top inflammation‐related biological processes of fucoidan against CG were revealed.

Gene ontology enrichment analysis aimed to identify all biological processes (BPs), cellular components (CCs), and molecular functions (MFs) of these core targets. By using adjusted *p* value < 0.05 and *q* value < 0.05, those 993 GO‐enriched terms were harvested (detailed in Table S2). The top 20 immunologic function–related terms were screened and highlighted in Figure 3, indicating that core targets exerted important action in immunoregulation for managing CG. Meanwhile, the top 20 GO terms associated with inflammation are shown in Figure 4, manifesting that core targets mediated a potential role in regulation of inflammatory stress for managing CG.

### 3.4 KEGG‐Based Enrichment Analysis Findings

**FIGURE 5:**
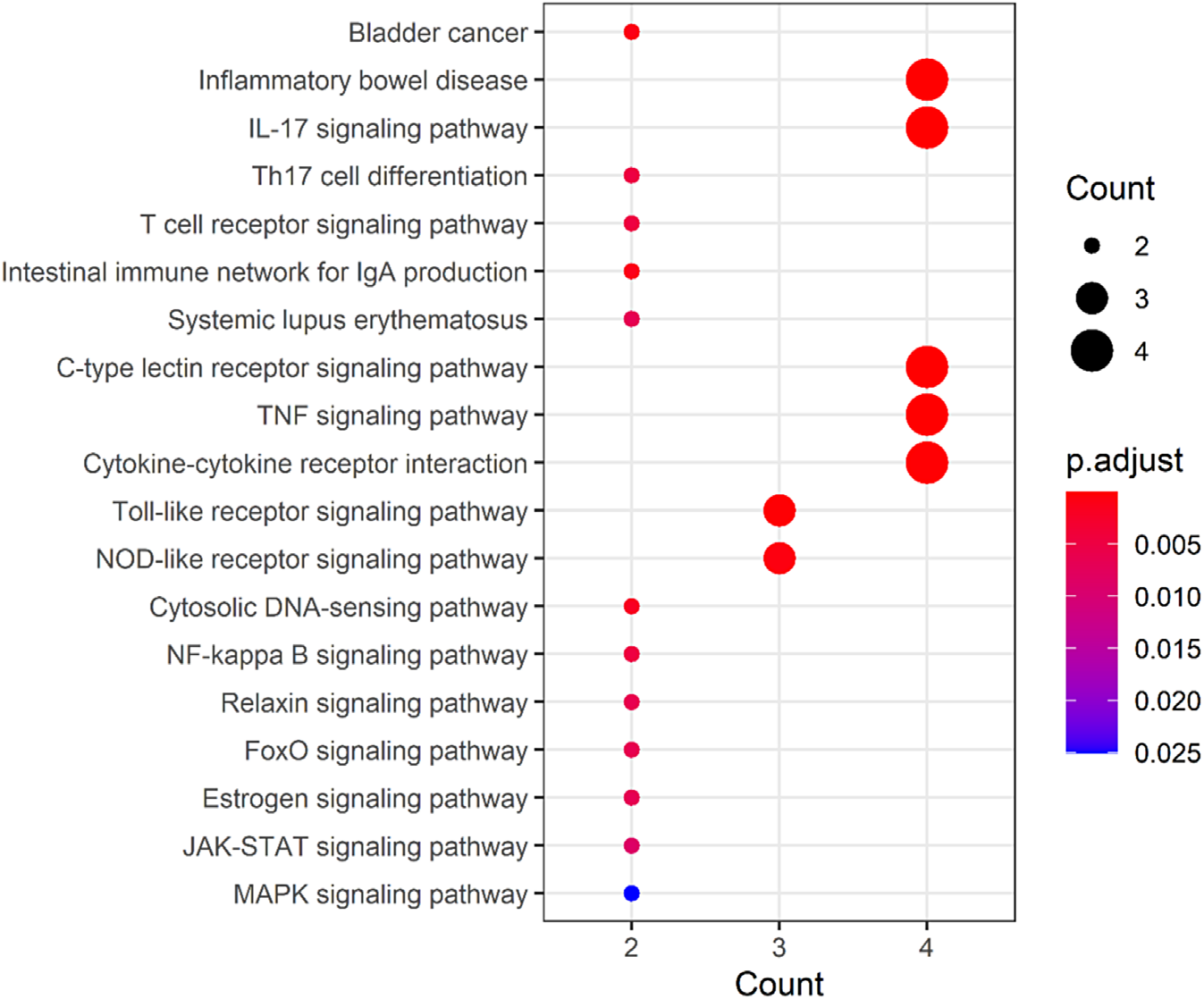
Current KEGG‐based enrichment analysis uncovered these top anti‐CG signaling pathways of fucoidan against CG.

KEGG enrichment analysis aimed to reveal the pharmacological mechanisms of fucoidan against CG. By utilizing adjusted *p* value < 0.05 and *q* value < 0.05, a total of 65 signaling pathways were enriched and identified accordingly, as revealed in Table S3. In detail, these anti‐CG molecular pathways were mainly involved in bladder cancer, inflammatory bowel disease, IL‐17 signaling pathway, Th17 cell differentiation, T‐cell receptor signaling pathway, intestinal immune network for IgA production, systemic lupus erythematosus, C‐type lectin receptor signaling pathway, TNF signaling pathway, cytokine–cytokine receptor interaction, Toll‐like receptor signaling pathway, nucleotide‐binding and oligomerization domain (NOD)‐like receptor signaling pathway, cytosolic DNA‐sensing pathway, nuclear factor kappa B (NF‐κB) signaling pathway, relaxin signaling pathway, Forkhead box O (FoxO) signaling pathway, estrogen signaling pathway, Janus kinase/signal transducer and activator of transcription (JAK/STAT) signaling pathway, and mitogen‐activated protein kinase (MAPK) signaling pathway (Figure 5).

### 3.5 Integrative Bioinformatics Findings

**FIGURE 6:**
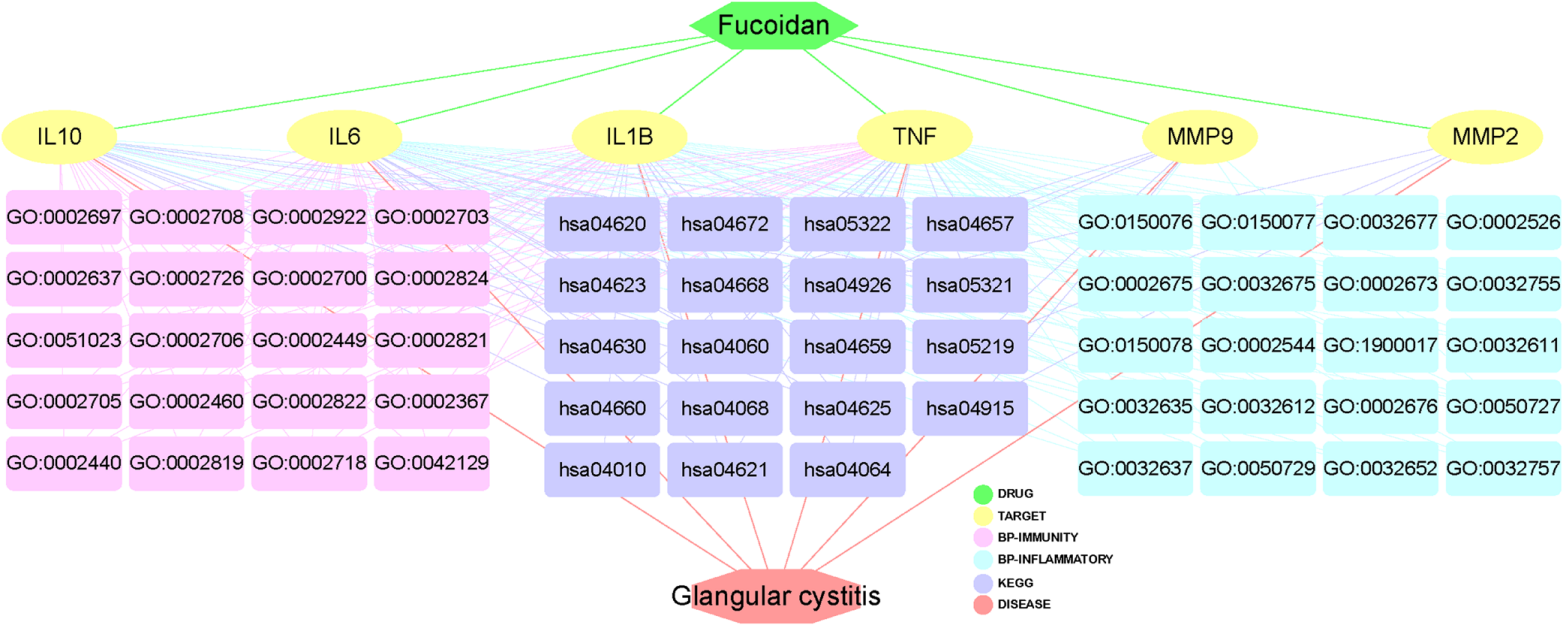
The interaction network diagram from all bioinformatics findings to reveal the fucoidan‐anti‐CG targets and mechanisms were characterized comprehensively.

Furthermore, all these bioinformatics data in this research were integrated for correlative visualization. Interestingly, an integrative network map including fucoidan‐target‐GO‐KEGG‐CG connections is plotted and detailed in Figure 6.

## 4 Discussion

By utilizing integrated network pharmacology analysis in this study, we identified all 16 intersection genes of fucoidan and CG, and a total of 6 core targets of fucoidan against CG were screened and ascertained. GO and KEGG enrichment analysis data uncovered that fucoidan exerted anti‐CG actions might be related to mainly modulating the biological processes of inflammatory stress, immunologic reaction, and immunoinfiltration, including the IL‐17 signaling pathway, Th17 cell differentiation, T‐cell receptor signaling pathway, TNF signaling pathway, cytokine–cytokine receptor interaction, Toll‐like receptor signaling pathway, and NF‐kappa B signaling pathway. These network pharmacology‐based findings could pharmacologically reveal the anti‐CG potentials of fucoidan predominantly through inflammation‐suppressing, immunoinfiltration‐reducing, and immunity‐enhancing actions. To exhibit more details, we aimed to explicate core targets in fucoidan against CG, as these genes might be the fucoidan‐anti‐CG pharmacological targets after experimental and clinical validation. IL‐6, a keystone cytokine, can play a broad action on the immune system, and cytokine storm symptoms based on its pro‐inflammatory property (Barrett 2024). Mounting studies indicate that IL‐6 plays a key role in modulating the homeostasis between regulatory T cells and Th17 cells, thus suppression of IL‐6 expression contributing to the therapy of inflammatory disorders (Kimura and Kishimoto 2010). It is clinically reported that elevated IL‐6 expressions were found in blood and tissue samples in CG patients, suggesting that IL‐6 may be a potential target for CG treatment (Qu et al. 2018). TNF, a pleiotropic factor, may regulate the functions of the immune system, cell survival, cell proliferation, and metabolism (Varfolomeev and Vucic 2018). TNF can induce the activation of effector T cells for proliferation, while cell apoptosis in high effector T‐cell activity is found by TNF action (Mehta, Gracias, and Croft 2018). As showed in clinical observation, TNF‐associated apoptosis‐triggering ligand may be responsible for the pathogenesis of interstitial cystitis (Kutlu et al. 2010). IL‐1B, a crucial mediator of inflammatory stress, can aggravate tissue impairment when chronic disease or acute injury persistently occurs (Lopez‐Castejon and Brough 2011). Cyclophosphamide‐induced severe hemorrhagic cystitis is detected with significantly elevated IL‐1B cytokine expression in vivo (Mousa et al. 2022). MMP‐9 may be involved in immune and inflammation responses because it can activate different cytokines and chemokines (Vafadari, Salamian, and Kaczmarek 2016). MMP‐9 may modulate the biological response to inflammatory stress after tissue damage (Wang et al. 2013). Notably, increased urinary contents of MMP‐9 and neutrophil gelatinase‐associated lipocalin in children with acute cystitis were reported clinically (Hatipoglu et al. 2011). IL‐10, an anti‐inflammatory cytokine, exerts an important role in regulating inflammatory infiltration and controlling adaptive immune responses that can induce tissue injury (Ouyang and O'Garra 2019). It is found that IL‐10 exhibits inhibition of antibody response in bladder impairment during infection (Choi and Abraham 2016). MMP‐2 is proven with the cytokine secretion and inflammatory process through being activated functionally (Ribeiro Vitorino et al. 2023). MMP‐2 is involved in the epigenetic onset of chronic cystitis in vivo via DNA methylation changes (Choi et al. 2013). These reference reports suggest that reduction of inflammatory response, cytokine infiltration, and enhancement of immune capability of fucoidan against CG are achieved. However, current limitations have been found in this bioinformatics study. Molecular docking imitation analysis should be determined for revealing the binding features between fucoidan and core target proteins in CG. Furthermore, further experimental or clinical validation needs to be performed for determining the pharmacological activities of fucoidan action to treat CG.

## 5 Conclusion

Collectively, current bioinformatics findings from this research highlight the anti‐CG potentials of fucoidan, characterizing detailed core targets and molecular mechanisms of fucoidan against CG prior to further validation. These results primarily exhibit that fucoidan may be a beneficial candidate for treating CG.

## Author Contributions

**Qingting Chen:** conceptualization (equal), data curation (equal), formal analysis (equal), methodology (equal), resources (equal). **Jie Mo:** data curation (equal), methodology (equal), resources (equal), software (equal), visualization (equal). **Yu Li:** conceptualization (equal), methodology (equal), resources (equal), software (equal), validation (equal), visualization (equal). **Li Gao:** investigation (equal), methodology (equal), project administration (equal), supervision (equal), validation (equal), writing – original draft (equal). **Ka Wu:** funding acquisition (equal), investigation (equal), project administration (equal), supervision (equal), writing – original draft (equal), writing – review and editing (equal).

## Conflicts of Interest

The authors declare no conflicts of interest.

## Supporting information


**Table S1.** Top six genes in network interactions ranked by degree method analysis.

**Table S2.** Integrated information in GO‐based annotation data.

**Table S3.** Integrated information in KEGG‐based enrichment data.

## Data Availability

The data that support the findings of this study are available upon request from the corresponding author.

## References

[fsn34560-bib-0001] Abdel Magied, M. H. , A. M. Badreldin , and S. W. Leslie . 2024. “Cystitis Cystica and Cystitis Glandularis.” In StatPearls. Treasure Island, FL: StatPearls Publishing. 35881730

[fsn34560-bib-0002] Bai, S. J. , X. B. Chen , and T. B. Zeng . 2023. “Treatment Progress of Cystitis Glandularis.” Asian Journal of Surgery 46, no. 6: 2444. 36581545 10.1016/j.asjsur.2022.12.060

[fsn34560-bib-0003] Barrett, D. 2024. “IL‐6 Blockade in Cytokine Storm Syndromes.” Advances in Experimental Medicine and Biology 1448: 565–572. 39117839 10.1007/978-3-031-59815-9_37

[fsn34560-bib-0004] Catarino, M. D. , A. M. S. Silva , and S. M. Cardoso . 2018. “Phycochemical Constituents and Biological Activities of *Fucus* spp.” Marine Drugs 16: 249. 30060505 10.3390/md16080249 PMC6117670

[fsn34560-bib-0005] Choi, H. W. , and S. N. Abraham . 2016. “Why Serological Responses During Cystitis Are Limited.” Pathogens 5, no. 1: 19. 26907352 10.3390/pathogens5010019 PMC4810140

[fsn34560-bib-0006] Choi, I. S. , K. Yu , J. Kim , et al. 2013. “Alterations in Deoxyribonucleic Acid (DNA) Methylation Patterns of Calca, Timp3, Mmp2, and Igf2r Are Associated With Chronic Cystitis in a Cyclophosphamide‐Induced Mouse Model.” Urology 82, no. 253: e9–e15. 23806407 10.1016/j.urology.2013.04.010 PMC3697025

[fsn34560-bib-0007] Dornelas‐Filho, A. F. , V. B. M. Pereira , D. V. T. Wong , et al. 2018. “Neutrophils Contribute to the Pathogenesis of Hemorrhagic Cystitis Induced by Ifosfamide.” International Immunopharmacology 62: 96–108. 29990699 10.1016/j.intimp.2018.06.031

[fsn34560-bib-0008] Ghallab, D. S. , R. S. Ibrahim , M. M. Mohyeldin , and E. Shawky . 2024. “Marine Algae: A Treasure Trove of Bioactive Anti‐Inflammatory Compounds.” Marine Pollution Bulletin 199: 116023. 38211540 10.1016/j.marpolbul.2023.116023

[fsn34560-bib-0009] Hatipoglu, S. , E. Sevketoglu , A. Gedikbasi , et al. 2011. “Urinary MMP‐9/NGAL Complex in Children With Acute Cystitis.” Pediatric Nephrology 26: 1263–1268. 21556719 10.1007/s00467-011-1856-3

[fsn34560-bib-0010] Kimura, A. , and T. Kishimoto . 2010. “IL‐6: Regulator of Treg/Th17 Balance.” European Journal of Immunology 40, no. 7: 1830–1835. 20583029 10.1002/eji.201040391

[fsn34560-bib-0011] Kohl, M. , S. Wiese , and B. Warscheid . 2011. “Cytoscape: Software for Visualization and Analysis of Biological Networks.” Methods in Molecular Biology 696: 291–303. 21063955 10.1007/978-1-60761-987-1_18

[fsn34560-bib-0012] Kutlu, O. , E. Akkaya , I. T. Koksal , et al. 2010. “Importance of TNF‐Related Apoptosis‐Inducing Ligand in Pathogenesis of Interstitial Cystitis.” International Urology and Nephrology 42, no. 2: 393–399. 19705295 10.1007/s11255-009-9632-z

[fsn34560-bib-0013] Li, R. , Y. Li , X. Liang , L. Yang , M. Su , and K. P. Lai . 2021. “Network Pharmacology and Bioinformatics Analyses Identify Intersection Genes of Niacin and COVID‐19 as Potential Therapeutic Targets.” Briefings in Bioinformatics 22: 1279–1290. 33169132 10.1093/bib/bbaa300 PMC7717147

[fsn34560-bib-0014] Lopez‐Castejon, G. , and D. Brough . 2011. “Understanding the Mechanism of IL‐1β Secretion.” Cytokine & Growth Factor Reviews 22, no. 4: 189–195. 22019906 10.1016/j.cytogfr.2011.10.001 PMC3714593

[fsn34560-bib-0015] Lu, S. , X. Sun , Z. Zhou , et al. 2023. “Mechanism of Bazhen Decoction in the Treatment of Colorectal Cancer Based on Network Pharmacology, Molecular Docking, and Experimental Validation.” Frontiers in Immunology 14: 1235575. 37799727 10.3389/fimmu.2023.1235575 PMC10548240

[fsn34560-bib-0016] Mehta, A. K. , D. T. Gracias , and M. Croft . 2018. “TNF Activity and T Cells.” Cytokine 101: 14–18. 27531077 10.1016/j.cyto.2016.08.003 PMC5305780

[fsn34560-bib-0017] Mousa, A. M. , K. S. Allemailem , F. A. Alhumaydhi , et al. 2022. “Cytoprotective Antioxidant, Anti‐Inflammatory, and Antifibrotic Impact of Celery Seed Oil and Manuka Honey Against Cyclophosphamide‐Induced Cystitis in Rabbits.” Evidence‐based Complementary and Alternative Medicine 2022: 2863023. 35341158 10.1155/2022/2863023 PMC8947928

[fsn34560-bib-0018] Noor, F. , M. Tahir Ul Qamar , U. A. Ashfaq , A. Albutti , A. S. S. Alwashmi , and M. A. Aljasir . 2022. “Network Pharmacology Approach for Medicinal Plants: Review and Assessment.” Pharmaceuticals 15, no. 5: 572. 35631398 10.3390/ph15050572 PMC9143318

[fsn34560-bib-0019] Ouyang, W. , and A. O'Garra . 2019. “IL‐10 Family Cytokines IL‐10 and IL‐22: From Basic Science to Clinical Translation.” Immunity 50, no. 4: 871–891. 30995504 10.1016/j.immuni.2019.03.020

[fsn34560-bib-0020] Pan, Q. , K. Wu , J. Tan , Y. Li , X. Liang , and M. Su . 2021. “Anti‐Neoplastic Characteristics and Potential Targets of Calycosin Against Bisphenol A‐Related Osteosarcoma: Bioinformatics Analysis.” Bioengineered 12: 4278–4288. 34311656 10.1080/21655979.2021.1956401 PMC8806932

[fsn34560-bib-0021] Qu, Y. , X. Chen , Y. Cui , W. He , and G. Wang . 2018. “Changes of Bladder Mucosal Inflammatory Factors and Prognosis in Cystitis Glandularis.” International Journal of Clinical and Experimental Pathology 11, no. 7: 3591–3597. 31949738 PMC6962861

[fsn34560-bib-0022] Ribeiro Vitorino, T. , A. F. do Prado , S. B. de Assis Cau , and E. Rizzi . 2023. “MMP‐2 and Its Implications on Cardiac Function and Structure: Interplay With Inflammation in Hypertension.” Biochemical Pharmacology 215: 115684. 37459959 10.1016/j.bcp.2023.115684

[fsn34560-bib-0023] Ronghua, W. , Z. Ji , L. Gang , Z. Yun , and N. Xubiao . 2024. “Cystitis Glandularis With Concomitant Crohn's Disease Leading to a Paroxysm of Crohn's Disease With Ulcerated External Iliac Vessels.” BMC Urology 24, no. 1: 89. 38632572 10.1186/s12894-024-01470-3 PMC11022458

[fsn34560-bib-0024] Sinanoglu, O. , A. N. Yener , S. Ekici , A. Midi , and F. B. Aksungar . 2012. “The Protective Effects of Spirulina in Cyclophosphamide Induced Nephrotoxicity and Urotoxicity in Rats.” Urology 80: 1392–1392.e6. 10.1016/j.urology.2012.06.053 22951000

[fsn34560-bib-0025] Takizawa, N. , T. Matsuzaki , T. Yamamoto , et al. 2016. “Novel Strategy for Cystitis Glandularis: Oral Treatment With Cyclooxygenase‐2 Inhibitor.” International Journal of Urology 23, no. 8: 706–708. 27238955 10.1111/iju.13121

[fsn34560-bib-0026] Vafadari, B. , A. Salamian , and L. Kaczmarek . 2016. “MMP‐9 in Translation: From Molecule to Brain Physiology, Pathology, and Therapy.” Journal of Neurochemistry 139: 91–114. 10.1111/jnc.13415 26525923

[fsn34560-bib-0027] Varfolomeev, E. , and D. Vucic . 2018. “Intracellular Regulation of TNF Activity in Health and Disease.” Cytokine 101: 26–32. 27623350 10.1016/j.cyto.2016.08.035

[fsn34560-bib-0028] Wang, H. J. , M. H. Pui , Y. Guo , et al. 2016. “Preliminary Study of Diffusion‐Weighted MRI in the Preoperative Diagnosis of Cystitis Glandularis.” Clinical Radiology 71: 937–937.e4. 10.1016/j.crad.2016.05.008 27320827

[fsn34560-bib-0029] Wang, X. , Y. Y. Yu , S. Lieu , et al. 2013. “MMP9 Regulates the Cellular Response to Inflammation After Skeletal Injury.” Bone 52, no. 1: 111–119. 23010105 10.1016/j.bone.2012.09.018 PMC3513654

[fsn34560-bib-0030] Yi, X. , H. Lu , Y. Wu , et al. 2014. “Cystitis Glandularis: A Controversial Premalignant Lesion.” Oncology Letters 8: 1662–1664. 25202387 10.3892/ol.2014.2360 PMC4156188

[fsn34560-bib-0031] Zhang, N. , M. Xue , T. Sun , J. Yang , Z. Pei , and K. Qin . 2022. “Fucoidan as an Autophagy Regulator: Mechanisms and Therapeutic Potentials for Cancer and Other Diseases.” Nutrition and Cancer 74, no. 5: 1568–1579. 34477470 10.1080/01635581.2021.1973045

[fsn34560-bib-0032] Zhou, R. , K. Wu , M. Su , and R. Li . 2019. “Bioinformatic and Experimental Data Decipher the Pharmacological Targets and Mechanisms of Plumbagin Against Hepatocellular Carcinoma.” Environmental Toxicology and Pharmacology 70: 103200. 31158732 10.1016/j.etap.2019.103200

